# Development of Microchip Isotachophoresis Coupled with Ion Mobility Spectrometry and Evaluation of Its Potential for the Analysis of Food, Biological and Pharmaceutical Samples

**DOI:** 10.3390/molecules26206094

**Published:** 2021-10-09

**Authors:** Jasna Hradski, Marta Ďuriš, Roman Szucs, Ladislav Moravský, Štefan Matejčík, Marián Masár

**Affiliations:** 1 Department of Analytical Chemistry, Faculty of Natural Sciences, Comenius University in Bratislava, 84215 Bratislava, Slovakia; hradski1@uniba.sk (J.H.); duris48@uniba.sk (M.Ď.); roman.szucs@uniba.sk (R.S.); 2 Department of Experimental Physics, Mathematics, Physics and Informatics, Comenius University in Bratislava, 84248 Bratislava, Slovakia; moravsky1@uniba.sk (L.M.); matejcik1@uniba.sk (Š.M.)

**Keywords:** microchip isotachophoresis, ion mobility spectrometry, carboxylic acids, foodstuffs, body fluids, pharmaceuticals

## Abstract

An online coupling of microchip isotachophoresis (µITP) with ion mobility spectrometry (IMS) using thermal evaporation interface is reported for the first time. This combination integrates preconcentration power of the µITP followed by unambiguous identification of trace compounds in complex samples by IMS. Short-chain carboxylic acids, chosen as model analytes, were first separated by the µITP in a discontinuous electrolyte system at pH 5–6, and subsequently evaporated at 130 °C during their transfer to the IMS analyzer. Various parameters, affecting the transfer of the separated sample components through the evaporation system, were optimized to minimize dispersion and loss of the analytes as well as to improve sensitivity. The following analytical attributes were obtained for carboxylic acids in the standard solutions: 0.1–0.3 mg L^−1^ detection limits, 0.4–0.9 mg L^−1^ quantitation limits, linear calibration range from the quantitation limit to 75 mg L^−1^, 0.2–0.3% RSD of the IMS response and 98–102% accuracy. The analytical potential of the developed µITP-IMS combination was demonstrated on the analysis of various food, pharmaceutical and biological samples, in which the studied acids are naturally present. These include: apple vinegar, wine, fish sauce, saliva and ear drops. In the real samples, 0.3–0.6% RSD of the IMS response and 93–109% accuracy were obtained.

## 1. Introduction

Food safety is the major focus of food analysis because of the known impact of the diet on human health. Food analysis involves the determination of a wide range of compounds of diverse physicochemical properties. Carboxylic acids play an important role in the food industry because of their antimicrobial activity and effect on the organoleptic properties of food, mainly taste and aroma, as well as its texture and overall quality [1]. Carboxylic acids occur naturally and as a result of fermentation in fruits and fruit juices, in dairy products, and also in wine and vinegar. Some carboxylic acids are used as food preservatives [2]. In addition to food, pharmaceuticals also have to be monitored for content of carboxylic acids which can be present as counter ions, process-related impurities and degradation products as they often represent key functional groups in active ingredients. Acetic acid is used as an active pharmaceutical ingredient to treat an outer ear infection by stopping the growth of bacteria and fungus [3]. Carboxylic acids are also an important part of biological processes as they are involved in various metabolite pathways as intermediates or end products. Accumulation of certain carboxylic acids in urine can indicate an organic acid disorder, e.g., an inherited metabolic disorder, propionic acidemia [4].

Carboxylic acids are present at different concentration levels in complex food, biological, pharmaceutical or environmental samples [4,5]; therefore, the development of advanced analytical methods and strategies for their determination is needed. Chromatographic techniques, i.e., gas chromatography and liquid chromatography combined with mass spectrometry are often used for this purpose [6]. With the advance of green analytical chemistry, miniaturization, automation and reduction in the use of toxic solvents play a key role in the development of modern analytical instrumentation. With this in mind, separation analytical techniques, enabling multi-component analysis, are preferred over single analyte applications [7]. Miniaturized analogs of conventional separation techniques can reduce the financial cost of analysis, speed up the analytical process, reduce the amount of the analyzed sample as well as the amount of waste produced. Various conventional separation techniques have already been miniaturized. A miniaturized version of capillary electrophoresis (CE), microchip electrophoresis (MCE), was successfully applied to the analysis of a wide range of compounds, e.g., small inorganic and organic ions, amino acids, pharmaceuticals and proteins [8,9]. However, shorter separation path and inner dimensions of the separation channels resulting from the miniaturization, lead to limited separation capacity and lower detection sensitivity of the MCE. This limits the applicability of MCE in the analysis of complex samples. For these reasons, MCE often requires the use of sample pretreatment prior to the analysis of complex samples and/or selective detection and/or identification technique. Ion mobility spectrometry (IMS) is a sensitive and fast separation and identification technique, which has been applied to the analysis of food, environmental and biological samples [6,10,11]. Even though IMS can be used as a stand-alone analytical technique, it is often used in combination with other separation techniques, e.g., gas chromatography, liquid chromatography or CE to increase its peak capacity and identification ability [12]. In coupling with mass spectrometry, it is used as a separation technique to improve its sensitivity [13].

MCE and IMS are based on a similar separation principle and due to their similarities, IMS was also called gaseous electrophoresis [14]. The combination of these two techniques into a two-dimensional (2D) system is advantageous because it combines the separation potential of MCE and the identification potential of IMS. In this way, problems associated with the identification of analytes present in complex samples after their MCE separation can be eliminated. At the same time, a significant increase in resolution in comparison to stand-alone IMS can be obtained. The biggest obstacle in combining these two techniques is the necessity to transfer analytes from liquid to the gaseous phase. Therefore, only a few attempts to combine IMS with CE [13,15,16,17], MCE [18] or chip-based electrochromatography [19] were made.

Even though zone electrophoresis, used for the combination of CE and MCE with IMS [13,15,16,17,18], is the most widely used electrophoretic technique, isotachophoresis (ITP) also emerges as a suitable electro-kinetic separation technique. Due to its ability to concentrate the analytes, it can either be used as a stand-alone technique or in combination with some other electroseparation techniques. ITP is implemented by two different electrolytes—the leading electrolyte (LE) and the terminating electrolyte (TE), and the sample is introduced between them. During analysis, sample components having effective mobilities in the range defined by the effective mobilities of the leading and terminating ions are separated and arranged in the order of decreasing effective mobilities. After reaching an ITP steady-state, analytes having different effective mobilities will form discrete zones with sharp boundaries between them. Based on the Kohlrausch regulating function, the concentration of the analytes in their own zones is constant and they migrate with the same velocity [20]. ITP has also been implemented on the microchip (microchip isotachophoresis; µITP), and combined with various detection techniques, e.g., conductivity detection [20] and surface enhanced Raman spectrometry [21]. It has been applied to the analysis of food and pharmaceutical samples.

In this paper, a novel hyphenation of µITP and IMS for the separation and determination of carboxylic acids (acetic acid, propionic acid, butyric acid, valeric acid and caproic acid) in various food, pharmaceutical and biological samples is presented.

## 2. Results and Discussion

### 2.1. Optimization of µITP Separation Conditions

As mentioned above, two different electrolytes need to be employed for the µITP separation. The selection of the electrolyte composition is dependent on the physicochemical properties of sample components being analyzed, mainly on their pKa values and ionic mobilities [22]. As part of the electrolyte optimization process, the fact that electrolyte components will be transferred to the IMS analyzer along with the separated sample components also has to be considered.

Effective mobilities of the separated sample components depend on the pH of the LE. In this work, µITP separations of six carboxylic acids, with experimentally confirmed pKa values in the range of 3.8 to 4.9 [23], were performed using the LE with pH 5.0. In addition, only ions having effective mobilities in the mobility interval defined by the leading ion and the terminating ion will be separated during µITP separation. Chloride was selected as a leading ion because its effective mobility is higher than the effective mobility of formate, the most mobile analyte. To maintain the constant pH of the LE during the separation, ε-amino-n-caproate was used as a counterion in the LE. 4-Morpholineethanesulfonate, having lower effective mobility than all the analytes, was used as a terminating ion (for more details about the composition of the LE and TE solutions see Section 3.1).

**Figure 1:**
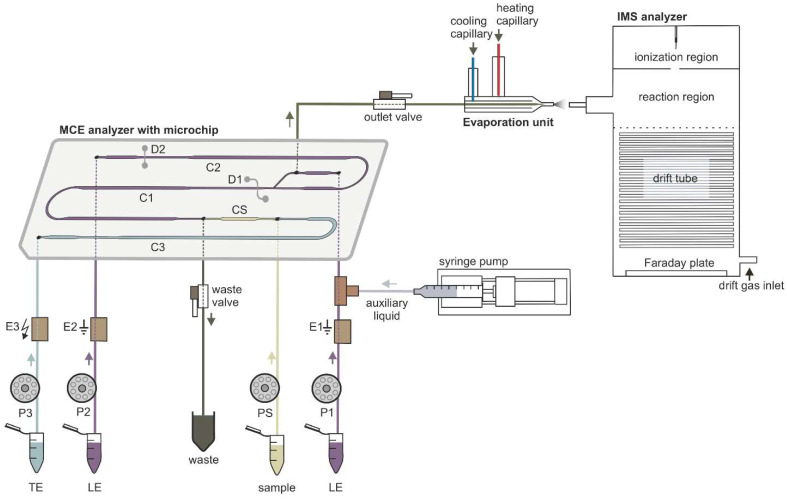
Schematic diagram of the instrumentation used for µITP-IMS analysis. P1, P2, P3, PS, peristaltic micropumps for filling microchip channels with LE (channels C1 and C2), TE (channel C3) and sample (channel CS) solutions; E1, E2, ground electrodes placed at the end of channels C1 and C2, respectively; E3, high voltage electrode placed at the end of channel C3; D1, D2, integrated conductivity sensors placed in channels C1 and C2, respectively.

The separations were performed under suppressed hydrodynamic flow on the microchip. During the separation, only one inlet to the microchip channels, used to introduce auxiliary liquid into the separation system, was open. Accordingly, only one outlet was kept open during the separation, which was used to transfer the separated sample components from the microchip to the evaporation unit (see Figure 1 for more details). Due to the suppressed hydrodynamic flow the electroosmotic flow (EOF) had to be eliminated in order to avoid dispersion [24]. In this context, the inner walls of the microchip channels were coated with methylhydroxyethylcellulose (MHEC), which acted as an EOF suppressor (for more details about the coating process see Section 3.4).

µITP separations were monitored by a conductivity detector, which was integrated directly onto the microchip. The separations were performed under a constant current of 20 μA, and before the sample components reached the conductivity detector the current was changed to 10 μA. In this way, approximately two times longer zone lengths (based on the ratio of the currents being applied) were observed for the sample components [20].

### 2.2. Optimization of µITP-IMS Transfer Parameters

To perform the µITP-IMS analysis, it was necessary to transfer the separated components from the first dimension (µITP) to the second dimension (IMS) with minimal dispersion. To achieve this, auxiliary liquid was applied to the microchip in the opposite direction to the isotachophoretic migration of the analytes on the microchip (for more details see Section 3.4. and Figure 1). Composition of the auxiliary liquid and its physicochemical properties, e.g., conductivity and pH, can significantly affect the µITP separation, and zone dispersion during the transfer as well as IMS response sensitivity. Various auxiliary liquids were tested including both electrolytes used for the µITP separation. A 10% (*v/v*) TE was found to be an optimal auxiliary liquid because it fulfilled the requirement of low dispersion transfer of the separated components from the microchip to the IMS analyzer. In addition, the chosen auxiliary liquid did not interfere with the µITP separation nor with the IMS response of the studied analytes.

**Figure 2:**
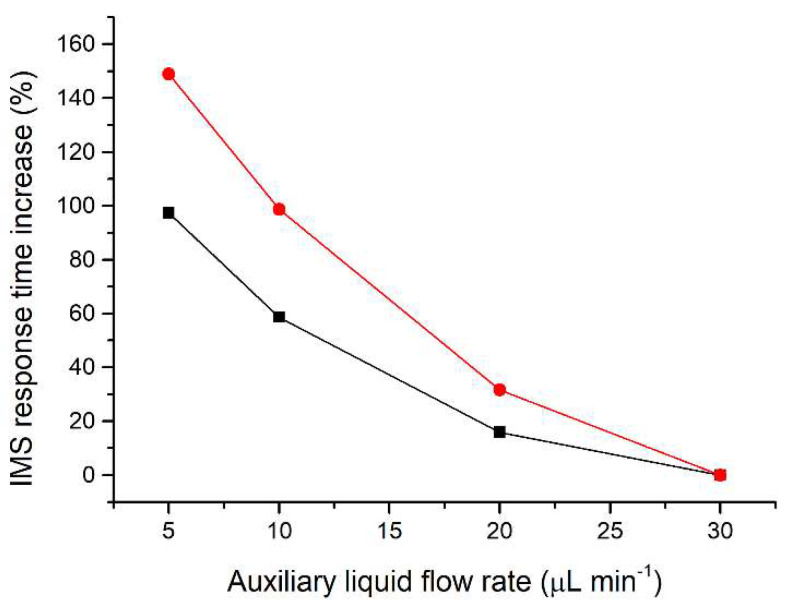
Impact of the auxiliary liquid flow rate on the IMS response time during µITP-IMS analysis. Expressed as percentage increase in the IMS response time for different flow rates compared to 30 µL min^−1^ for a two-component mixture of acetic acid (black line) and valeric acid (red line) at a 50 mg L^−1^ concentration.

Since the separated sample components are transferred together with the auxiliary liquid, effectively acting as a diluent, the flow rate of the auxiliary liquid is crucial to minimize the zone dispersion. The range of auxiliary liquid flow rates tested (5–30 µL min^−1^; Figure 2) was chosen to achieve compatibility with the velocity of the sample components during their separation on the microchip. For optimization of the flow rate of the auxiliary liquid a two-component mixture of acetic acid and valeric acid at a 50 mg L^−1^ concentration was used. These components were selected because they have different effective mobilities and thus create individual zones during the µITP separation. Additionally, acetic acid provides a very sensitive IMS response, while valeric acid has a lower IMS response, similar to other less volatile carboxylic acids used as the analytes. The investigated flow rates did not affect the µITP separation nor caused distortion of the ITP steady state zones, as monitored by the conductivity detector on the microchip. On the other hand, the flow rate affected the IMS response time of the sample components. From data presented in Figure 2, it is evident that with increasing flow rate of the auxiliary liquid the overall IMS response time for each sample component decreases. In the same way, the transfer time between the µITP separation and the IMS analysis was prolonged by the lower flow rate used. This led to a significantly higher dispersion of the separated zones of the analytes, as evident from the IMS response. In addition, approximately 30% increase in the total µITP-IMS analysis time was observed when 5 µL min^−1^ was used in comparison to 30 µL min^−1^. Therefore, a 30 µL min^−1^ flow rate of the auxiliary liquid was chosen for further experiments.

The transfer of the sample components from the µITP to the IMS analyzer also includes the change of phase in which the separated sample components are present, from liquid to gaseous. An evaporation unit [25] was connected directly to the MCE analyzer and therefore it could also affect the µITP separation. The temperature of the droplet stream exiting from the evaporation unit was optimized in the range of 110–170 °C [18]. The use of a lower temperature of the droplet stream resulted in insufficient evaporation of the separated sample components, whereas a higher temperature caused problems with the transfer, which affected µITP separation. The optimal temperature of the droplet stream, leading to a minimal impact on the µITP separation, was found to be 130 °C.

### 2.3. µITP-IMS Method Performance Parameters

**Figure 3:**
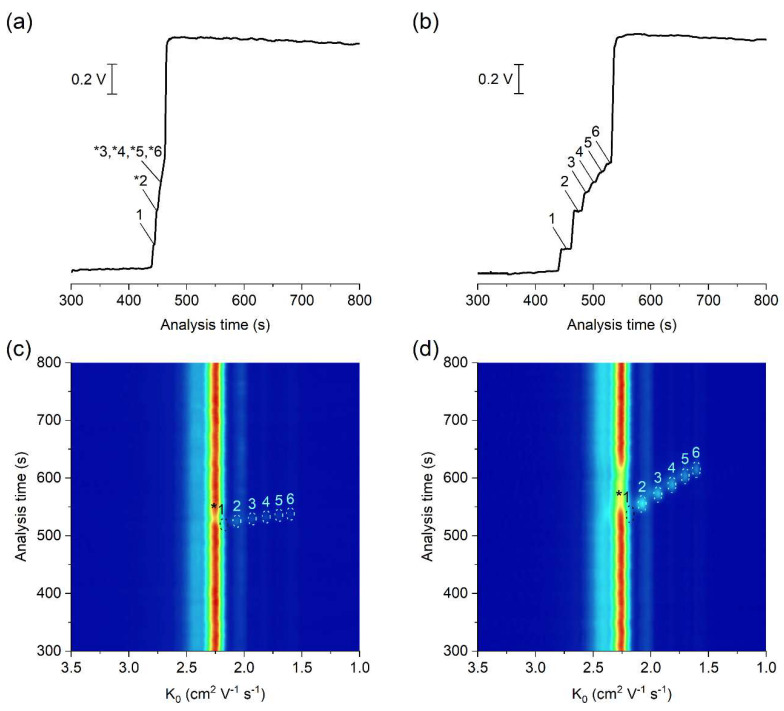
μITP-IMS analysis of standard solutions of C_1_–C_6_ carboxylic acids at (**a**,**c**) 10 mg L^−1^ and (**b**,**d**) 50 mg L^−1^. (**a**,**b**) Responses from conductivity detector integrated directly on the μITP microchip. (**c**,**d**) 2D plots from μITP-IMS analysis. Formic acid (1), acetic acid (2), propionic acid (3), butyric acid (4), valeric acid (5) and caproic acid (6). Auxiliary liquid: 10% TE; flow rate: 30 μL min^−1^. Other μITP-IMS parameters are given in Materials and Methods. * position of the analyte.

The potential of the developed µITP-IMS method was shown on the analysis of C_1_–C_6_ carboxylic acids (Figure 3). ITP with conductivity detection, as an universal type of detection, is used mainly for the determination of the analytes present at higher concentration levels in the sample (Figure 3b). In such case, provided that effective mobilities of the analytes are different, each of them will migrate in its own zone. Then, the zone length, i.e., the time needed for the analyte to pass through the conductivity detector, can be used as a quantitative parameter [20]. However, if the analytes are present at trace concentrations in the sample, they are unable to form their own zones, and migrate between a pair of fully developed zones [21]. In this case, a selective detection technique has to be used instead of universal conductivity detection (as evident from Figure 3a). IMS offers a greater advantage because, in addition to being used for detection, it can also selectively identify individual sample components. Reduced ion mobility (K_0_), used as a qualitative parameter, is influenced by the mass, charge and shape of the ion.

In this work, negative corona discharge ionization was used in the IMS. The reactant ion (K_0_ = 2.24 cm^2^ V^−1^ s^−1^; Figure 3c,d), which ionized the components via electron transfer reactions, was composed of O_2_^−^(H_2_O)_n_ [26]. After introducing the separated sample components into the IMS, a decrease in the intensity of reactant ions was observed on the 2D plot, while product ions with a K_0_ value characteristic of the individual carboxylic acids were formed (Figure 3c,d and Table 1). The K_0_ value of formic acid (2.17 cm^2^ V^−1^ s^−1^) was close to the K_0_ of reactant ions under the used IMS working conditions. Therefore, its unambiguous identification was practically impossible, and this analyte was not evaluated in standard solutions nor in real samples.

A possible impact of electrolytes, used for the µITP separation, on the IMS response was studied as well. After introduction of LE and/or TE mixed with the auxiliary liquid, only a slight reduction in the intensity of the reactant ion peak was observed. No new product ions were formed in comparison to the IMS response of drift gas.

Even though the K_0_ values are normalized to the standard temperature (273 K) and the pressure (760 torr) they are also affected by the composition of drift gas, electric field and moisture. Therefore, slight differences can be observed when comparing K_0_ values with the values reported in the literature [27]. In this work, the K_0_ values of carboxylic acids were nearly identical to those obtained under similar IMS conditions after their separation by zone electrophoresis on the microchip [18].

Several analytical parameters of the developed µITP-IMS method were evaluated for each of the carboxylic acids in the standard solutions, including sensitivity, linearity, accuracy and precision (Table 1). A standard procedure was used for calculation of the limit of detection (LOD) based on 3.3 times of the standard deviation of the blank (noise) to the slope of the calibration curve constructed from the peak height. The limit of quantitation (LOQ) was calculated based on 10 times of the standard deviation of the blank to the slope of the calibration curve. LODs ranged from 0.13 to 0.31 mg L^−1^ and LOQs ranged from 0.39 to 0.93 mg L^−1^ for studied carboxylic acids. Linearity of the method was investigated for the peak height and peak area from three repeated measurements at 8 and 13 concentration levels, respectively. The calibration solutions were injected in random order. The linear dynamic range for the peak height was from LOQ to 20 mg L^−1^ and for the peak area from LOQ to 75 mg L^−1^.

Accuracy was evaluated as percent recovery from determination of known concentration of the analyte in the sample diluent using the equations of regression lines listed in Table 1. The obtained accuracy in the range 98–102% for standard solutions is acceptable for the current stage of development of this technology. Precision of the method, evaluated as relative standard deviation (RSD) of the K_0_, ranged from 0.22% to 0.30% indicating good repeatability of the identification parameter.

### 2.4. Analysis of Food, Biological and Pharmaceutical Samples

**Figure 4:**
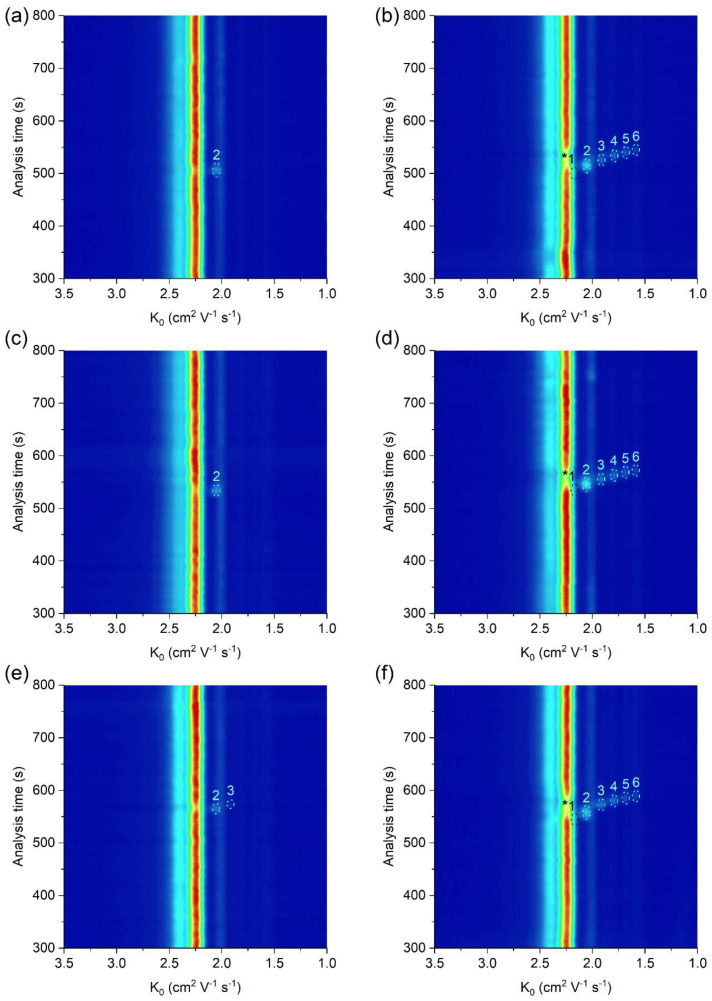
μITP-IMS analysis of (**a**) 4000-times diluted apple vinegar, (**b**) 4000-times diluted apple vinegar with addition of 20 mg L^−1^ of C_1_–C_6_ carboxylic acids, (**c**) 2000-times diluted ear drops, (**d**) 2000-times diluted ear drops with addition of 20 mg L^−1^ of C_1_–C_6_ carboxylic acids, (**e**) 10-times diluted saliva, (**f**) 10-times diluted saliva with addition of 20 mg L^−1^ of C_1_–C_6_ carboxylic acids. Formic acid (1), acetic acid (2), propionic acid (3), butyric acid (4), valeric acid (5) and caproic acid (6). Auxiliary liquid: 10% TE; flow rate: 30 μL min^−1^. Other μITP-IMS parameters are given in Materials and Methods. * position of the analyte.

The practical applicability of the μITP-IMS method was verified by the analysis of food (apple vinegar, wine, fish sauce), biological (saliva) and pharmaceutical (ear drops) samples, in which the presence of at least one acid from the homologous series of C_2_–C_6_ carboxylic acids was expected (Figure 4, Table 2). The sample preparation prior to the μITP-IMS analysis varied depending on the complexity of the sample and individual procedures are described in detail in Section 3.2. The dilution factor was estimated based on the expected concentration of the studied carboxylic acids in individual types of the samples found in the literature. A preliminary study with diluted samples was then performed, so that the IMS response of the analytes lies in the middle of the calibration range.

As a result of fermentation process, acetic acid is expected in wide range of food products. In apple vinegar, which is used as an acidifier, flavor enhancer and food preservative, mostly acetic acid was expected to be present. Concentration of acetic acid in the apple vinegar was determined to be 79.2 g L^−1^, while no other studied carboxylic acids were detected. Similar results were obtained in the study focused on the determination of organic acids in various vinegars [28]. Concentration of acetic acid in wine varies based on the wine production process [1], and it should be monitored as it significantly affects the organoleptic properties of wine. In the white wine analyzed, concentration of acetic acid was found to be 212.3 mg L^−1^. The presence of acetic acid was confirmed also in the fish sauce, produced by fish fermentation, at concentration of 901.2 mg L^−1^. This compares well with the content found in the literature [29].

From the studied carboxylic acids, mostly acetic acid was expected in saliva, as a result of various metabolite pathways in the human body. Acetic acid and propionic acid were detected in the analyzed saliva, as suggested in the literature [30]. The concentration of acetic acid was found to be 130.2 mg L^−1^ and the concentration of propionic acid was 44.7 mg L^−1^. Acetic acid is also often present in pharmaceuticals either as a counterion or active ingredient in antimicrobial products. The concentration of acetic acid in the analyzed ear drops was found to be 50.1 g L^−1^.

As evident, in most of the analyzed real samples only acetic acid was detected and determined. In order to show the practical applicability of the developed method for the simultaneous determination of all studied carboxylic acids, real samples spiked with a standard addition of C_1_–C_6_ carboxylic acids were analyzed (Figure 4b,d,f). K_0_ values of C_2_–C_6_ carboxylic acids in the analyzed real samples with standard addition were practically the same as those in the standard solutions, which implies a minimal sample matrix effect on the K_0_ values (Table 2). Based on accuracy data in Table 2, the developed µITP-IMS method was found to be suitable for the analysis of studied carboxylic acids in the samples of different origin.

## 3. Materials and Methods

### 3.1. Chemicals and Solutions

Chemicals of p.a. purity, used to prepare µITP electrolytes and standard solutions, were purchased from Sigma-Aldrich (Steinheim, Germany), Merck–Millipore (Darmstadt, Germany), Serva (Heidelberg, Germany) and Lachema (Brno, Czech Republic). The LE used for μITP separations consisted of 10 mmol L^−1^ hydrochloric acid and 50 mmol L^−1^ ε-amino-n-caproic acid at pH 5.0 and the TE consisted of 20 mmol L^−1^ 4-Morpholineethanesulfonic acid and 20 mmol L^−1^ L-histidine at pH 6.1. Standard solutions of carboxylic acids (formic acid, acetic acid, propionic acid, butyric acid, valeric acid and caproic acid) were prepared at a 1 g L^−1^ concentration. MHEC 30,000 (Serva), used as an EOF suppressor, was prepared at a 0.1% (*w/v*) concentration. Water purified through a Pro-PS system (Labconco, Kansas City, KS, USA) and then deionized by a Simplicity deionization unit (Millipore, Molsheim, France) was used for the preparation of the electrolyte, standard and sample solutions. The electrolyte and standard solutions were stored at 4 °C and used for a maximum of one week. The electrolytes were filtered through a 0.45 μm glass fiber filters (Merck–Millipore) prior to use.

### 3.2. Food, Biological and Pharmaceutical Samples

The analyzed food and pharmaceutical samples were purchased at a local store and pharmacy. Low-salt food and pharmaceutical samples (wine, apple vinegar and ear drops) were analyzed after simple pretreatment which included, filtration through a 0.45 µm glass fiber filter (Merck–Millipore). Fish sauce, having a high salt content of 230 g L^−1^, was first pretreated using micro-solid-phase extraction to eliminate excess of chloride. The extraction procedure was performed according to the literature [31].

Saliva was collected from a healthy adult volunteer, who refrained from eating, drinking, or performing any oral hygiene for 4 h prior to unstimulated saliva collection. The saliva sample was filtered through a 0.45 µm glass fiber filter (Merck–Millipore) after collection.

Prior to their injection on the microchip, the pretreated sample solutions were homogenized using vortex REAX 2000 (Heidolph Instruments, Schwabach, Germany), and appropriately diluted with deionized water.

### 3.3. µITP-IMS Instrumentation

The MCE analyzer, evaporation unit and IMS analyzer were connected in the way shown in the schematic diagram in Figure 1 to achieve online µITP-IMS coupling. The optimal parameters of the instrumentation used for the µITP-IMS analysis are summarized in Table 3.

The MCE analyzer used for µITP separations consisted of three parts—a microchip, an electrolyte unit and an electronic unit (Department of Analytical Chemistry, Comenius University in Bratislava). µITP separations were carried out on a poly(methyl methacrylate) microchip with coupled separation channels (IonChip^TM^ 3.0; Merck, Darmstadt, Germany). The microchip had integrated conductivity sensors, which were used for direct monitoring of the µITP separation. The inlets of the microchip channels were connected to the electrolyte unit via polyether ether ketone (PEEK) capillaries of 500 µm inner diameter (i.d.) (IDEX Health & Science, Wertheim, Germany). The electrolyte unit consisted of membrane driving electrodes (E1, E2, E3) and peristaltic micropumps (P1, P2, P3, PS), which were interconnected via fluorinated ethylene propylene capillaries of 500 µm i.d. (IDEX Health & Science). The membrane driving electrodes were used to eliminate interference caused by bubble generation during application of the driving current [24]. Peristaltic micropumps were used to transport solutions to the individual microchip channels. The rollers of the peristaltic micropumps automatically closed the microchip inlets after filling the microchip channels and thus ensured the elimination of hydrodynamic flow. Two shut-off valves (IDEX Health & Science) were used to achieve proper filling of the microchip channels and closing of the separation space. The waste valve (1 mm i.d., 10 μL volume) was placed on the waste capillary of the MCE analyzer. The outlet valve (0.5 mm i.d., 2.5 μL volume) was placed on the microchip outlet capillary (PEEK, 0.178 mm i.d.) and acted as a connection to the evaporation unit inlet capillary (PEEK, 0.254 mm i.d.). In this way outlet valve served for transfer of the analytes from the MCE analyzer to the evaporation unit. Auxiliary liquid was introduced to the microchip using a syringe pump (Cole-Parmer, Vernon Hills, IL, USA) with a 2.5 mL syringe (Hamilton, Bonaduz, Switzerland). An auxiliary liquid capillary (PEEK, 0.127 mm i.d.) connected syringe pump with a PEEK mixing tee (IDEX Health & Science), located between the E1 electrode and the microchip. The electronic unit was used as a source of stabilized electric current. It also included the measuring electronics of the contact conductivity detector and controlled the peristaltic micropumps operation.

The evaporation unit (Department of Experimental Physics, Faculty of Mathematics, Physics and Informatics, Comenius University in Bratislava) was used to connect the MCE analyzer to the IMS analyzer [25]. The evaporation unit consisted of three capillaries: an evaporation inlet capillary for delivering liquid sample to the evaporation unit, a cooling capillary and a heating capillary. The air flowing from the cooling capillary protected the evaporation unit inlet capillary from overheating, which could cause sample loss during the transfer of sample components from the MCE analyzer to the IMS analyzer. The air flowing from the heating capillary was mixed with air from the cooling capillary and liquid sample at the end of the evaporation inlet capillary, which resulted in evaporation of the sample. The outlet from the evaporation unit was placed in front of the IMS inlet capillary, and in this way evaporated sample was introduced into the IMS analyzer.

The IMS analyzer (Department of Experimental Physics, Comenius University in Bratislava in collaboration with MaSaTECH, Bratislava, Slovakia) operated in reverse gas flow mode; the drift gas inlet was at the end of drift tube and the gas outlet was behind the corona discharge. Atmospheric air purified by molecular sieve moisture trap (Agilent, Santa Clara, CA, USA) was used as a drift gas. Sample evaporated using the evaporation unit entered the reaction region where it was ionized. The IMS analyzer was equipped with a corona discharge ionization source. Ionized sample was then introduced into a drift tube, through a Bradbury–Nielsen shutter grid, which regulated the entry of the ions into the drift tube. Subsequently, the ions were separated in a drift tube and the signal of the ions was detected at the end of the drift tube on a Faraday plate and amplified by a current amplifier. The control unit was used for recording IMS spectra and setting the operating parameters. The IMS analyzer was calibrated using K_0_ of 2,6-di-*tert*-butyl pyridine [32].

### 3.4. Microchip Maintenance and Measurement Procedure

To suppress the EOF, the microchip channels were filled daily with a 0.1% (*w/v*) MHEC for 5 min prior to the first analysis. Afterwards, the channels were rinsed with deionized water for 10 min and subsequently filled with the electrolyte and sample solutions using peristaltic micropumps. During the filling procedure outlet valve was closed and the excess of solutions was removed from the microchip through the open waste valve. When the filling procedure ended, the waste valve was closed, and the outlet valve was opened to ensure transfer of all separated sample components from the microchip to the evaporation unit. Upon switching on the driving current, which was applied between the E3 and E1 electrodes, the anions were migrating from the sample channel towards the E1 electrode (anode). Simultaneously, the syringe pump was switched on and the auxiliary liquid was introduced onto the microchip in the direction opposite to the electrophoretic migration of the anions on the microchip. The auxiliary liquid along with the separated sample components exited from the microchip through the only open outlet, which was connected to the evaporation unit. At the outlet from the evaporation unit, the separated sample components were evaporated and subsequently syphoned into the reaction region of the IMS analyzer, where they underwent a reaction with the reactant ions. Further separation of ions continued in the drift tube and the detection took place on a Faraday plate.

Between the analyses, the microchip channels were only refilled with the electrolyte and sample solutions for 3 min. After the last analysis of the day, the channels were rinsed with 2% (*v/v*) aqueous detergent solution (Extran MA 02, Merck, Kenilworth, NJ, USA) for 5 min and deionized water for 10 min.

### 3.5. Data Processing

Conductivity data were recorded using MicroITP software (Merck, Kenilworth, NJ, USA), while IMS data, presented in the form of 2D plots, were recorded by IMS control software (MaSaTECH, Calgary, AB, Canada) and processed using Octave software (4.4.1). During creation of the 2D plots, spectra were treated using a moving average filter. Each point of the 2D heat map plot was characterized by the overall analysis time (including µITP separation time, transfer time and IMS drift time), the K_0_ value and intensity of the IMS response (expressed as ion yield detected by the IMS analyzer).

## 4. Conclusions

In this work, online µITP-IMS combination has been developed, tested and successfully applied to the analysis of complex biological, food and pharmaceutical samples. The coupling combined high preconcentration capabilities of µITP with sensitive IMS identification. The crucial point of such coupling is the transfer of separated analytes from the liquid to the gaseous phase. Optimization of transfer parameters, mainly the flow rate of the auxiliary liquid as well as the temperature for evaporation, led to minimal impact on the separation of the analytes and their quantification. Five carboxylic acids from the homologous series serving as model analytes were successfully separated and identified by the µITP-IMS coupling. Practical applicability of the developed approach was demonstrated by the analysis of five real samples of different origin. Acetic acid was determined in apple vinegar, wine, fish sauce, ear drops and saliva, while propionic acid was determined in saliva. A standard addition method was used to confirm the presence of carboxylic acids in the analyzed samples based on their K_0_ values. The developed approach can be beneficial for the determination of trace analytes, especially in the situations where µITP coupled with universal conductivity detection is failing to sufficiently separate and detect minor components.

## Figures and Tables

**Table 1:** Performance characteristics of the μITP-IMS method.

Analyte	LOD [mg L^−1^]	LOQ [mg L^−1^]	Equation of Regression Line	Correlation Coefficient	Accuracy [%] ^a^	K_0_ [cm^2^ V^−1^ s^−1^] (RSD [%]) ^a^
Acetic acid	0.13	0.39	y = 0.561x + 0.178	0.999	99.7–101.5	2.07 (0.23)
Propionic acid	0.15	0.46	y = 0.518x + 0.096	0.999	97.5–101.1	1.94 (0.22)
Butyric acid	0.19	0.58	y = 0.428x + 0.089	0.999	99.3–101.7	1.82 (0.27)
Valeric acid	0.23	0.70	y = 0.375x + 0.023	0.999	99.2–100.7	1.70 (0.30)
Caproic acid	0.31	0.93	y = 0.278x + 0.053	0.999	98.5–102.4	1.60 (0.29)

^a^ n = 3, at three concentration levels of the analytes: 5 mg L^−1^, 20 mg L^−1^ and 50 mg L^−1^.

**Table 2:** Results of the analysis of real samples.

Analyte	Concentration [mg L^−1^]	Accuracy [%] ^a^	K_0_ [cm^2^ V^−1^ s^−1^] (RSD [%]) ^a^
Acetic acid	79,221.5 ^b^; 212.3 ^c^; 901.2 ^d^; 130.2 ^e^; 50,124.5 ^f^	92.5–108.3	2.06 (0.40)
Propionic acid	44.7 ^e^	93.0–108.0	1.93 (0.29)
Butyric acid	n.d.	100.9–108.7	1.81 (0.50)
Valeric acid	n.d.	94.5–107.7	1.69 (0.58)
Caproic acid	n.d.	93.9–104.2	1.60 (0.31)

^a^ each sample analyzed in triplicate, 20 mg L^−1^ of the analytes added; ^b^ apple vinegar; ^c^ wine; ^d^ fish sauce; ^e^ saliva; ^f^ ear drops; n.d. not detected.

**Table 3:** Parameters of the instrumentation for the µITP-IMS analysis.

Instrument	Parameter	Setting
MCE analyzer	operating mode	ITP–anionic
	driving current	20 (10 at detection point) µA
	effective separation path (length × width × depth)	59 × 0.2–0.5 × 0.14–0.2 mm
Evaporation unit	flow rate	30 µL min^−1^
	temperature of droplet stream	130 °C
IMS analyzer	operating mode	negative
	sample gas flow rate	100 mL min^−1^
	shutter grid pulse width	100 µs
	drift gas flow rate	600 mL min^−1^
	drift field intensity	565 V cm^−1^
	drift tube temperature	100 °C
	operating pressure	600 mbar
	acquisition time	130 ms

## Data Availability

Not applicable.

## Sample Availability

Samples of the compounds are not available from the authors.
